# IP3R Channels in Male Reproduction

**DOI:** 10.3390/ijms21239179

**Published:** 2020-12-02

**Authors:** Xiaoning Zhang, Rongzu Huang, Yang Zhou, Wenwen Zhou, Xuhui Zeng

**Affiliations:** 1 Institute of Reproductive Medicine, Medical School, Nantong University, Nantong 226019, China; hrz1030607976@163.com (R.H.); zhouyangntu@163.com (Y.Z.); zww950820@163.com (W.Z.); 2 Institute of Life Science, Nanchang University, Nanchang 330031, China

**Keywords:** calcium signaling, IP3R, male reproduction, infertility, spermatogenesis, sperm function

## Abstract

As a second messenger in cellular signal transduction, calcium signaling extensively participates in various physiological activities, including spermatogenesis and the regulation of sperm function. Abnormal calcium signaling is highly correlated with male infertility. Calcium signaling is mainly regulated by both extracellular calcium influx and the release of calcium stores. Inositol 1,4,5-trisphosphate receptor (IP3R) is a widely expressed channel for calcium stores. After being activated by inositol 1,4,5-trisphosphate (IP3) and calcium signaling at a lower concentration, IP3R can regulate the release of Ca^2+^ from stores into cytoplasm, and eventually trigger downstream events. The closure of the IP3R channel caused by a rise in intracellular calcium signals and the activation of the calcium pump jointly restores the calcium store to a normal level. In this review, we aim to discuss structural features of IP3R channels and the underlying mechanism of IP3R channel-mediated calcium signaling and further focus on the research progress of IP3R expression and function in the male reproductive system. Finally, we propose key directions and strategies for research of IP3R in spermatogenesis and the regulation of sperm function to provide more understanding of the function and mechanism of IP3R channel actions in male reproduction.

## 1. Introduction

Ca^2+^ serves as a second messenger in cellular signal transduction to widely regulate many intracellular events, such as gene expression, synaptic transmission, and cellular death, differentiation and proliferation. Studies have shown that calcium signaling is also involved in spermatogenesis, sperm function, and fertilization, essential processes for male reproduction [1]. Calcium ions are stored in spermatogenic cells at various developmental stages, and also in Leydig and Sertoli cells [2]. The concentration of calcium in spermatogenic cells gradually increases from the early to late stages of spermatogenesis (spermatogonia < spermatocytes < spermatids < spermatozoa), and the concentration of calcium ions in seminiferous tubules also varies in different sections [2,3]. Calcium signaling in Leydig cells regulates the production of steroids through nuclear receptors and emergency response proteins mediated by a transcription cascade [4]. Abnormal calcium signaling can decrease testosterone levels, which results in abnormal spermatogenesis and even male infertility [5]. Some calcium-modulated or -binding proteins, such as calmodulin, are abundantly expressed in the testis [6]. The distinct distribution or oscillation of calcium levels and the expression of its regulatory proteins during spermatogenesis suggest that calcium signaling may play an essential role in spermatogenesis, in particular in the regulation of the growth and apoptosis of spermatogonia and spermatocytes [7,8]. Calcium signals also participate in the process of epididymal sperm maturation [9]. Studies have shown that some calcium channels maintain calcium homeostasis and regulate calcium signals, in turn affecting spermatogenesis. The treatment of mice with calcium channel inhibitors nifedipine and ethosuximide significantly blocked spermatogenesis at the elongating spermatid stage [10]. In addition, the expression of spermatogenesis-related genes *Cerm*, *Transition protein 2*, and *Protamine 2* were significantly upregulated by these inhibitors, which further implies that calcium signaling plays a crucial role in spermatogenesis [10]. The content of Ca^2+^ in semen from infertile patients with asthenozoospermia and varicocele was significantly lower than that in normal males [11,12], and some patients with idiopathic infertility had significantly lower calcium levels in seminal plasma than fertile males [13,14]. These studies suggested that reduced calcium levels in seminal plasma may be a key cause of male infertility, and calcium in seminal plasma may have an important regulatory effect on sperm function. Future studies are required to verify this role. There have been many studies showing that calcium signaling regulates mature sperm function, such as the sperm acrosome reaction, capacitation, chemotaxis, and motility, as well as fertilization [1,15]. The absence of the sperm-specific calcium channel CatSper inhibited the influx of extracellular calcium, which impaired the hyperactivated mobility of sperm and led to male infertility [16]. However, there remains a lack of evidence regarding the function of calcium signaling in the different subcellular units of sperm, and the function of other calcium regulatory proteins in sperm calcium signaling and male infertility remains unknown. Intracellular calcium signaling is mainly regulated by calcium efflux, influx, and store release. Calcium efflux is regulated by the plasma membrane Ca^2+^-ATPase [17,18] and Na^+^/Ca^2+^ exchanger [19,20]. Although research on calcium influx during spermatogenesis has been scarce, studies in recent years have analyzed the function and mechanism of the CatSper channel in mature sperm and showed that disrupted calcium influx in sperm was closely related to male infertility. Deletion or mutation of the main subunit of CatSper often causes male sterility [16,21,22]. Research into the role and mechanism of calcium storage regulation during spermatogenesis and sperm function remains incomplete. Calcium stores combined with other key factors play critical roles in calcium homeostasis and calcium signaling. Generally, the increase in cytosolic Ca^2+^ is regulated by inositol 1,4,5-trisphosphate receptors (IP3Rs), ryanodine receptor channels, transient-receptor-potential channels, cyclic-nucleotide-gated channels, transient-receptor-potential-vanilloid channels, the store-operated Ca^2+^-channel and CatSper channel [1]. A decrease in cytosolic Ca^2+^ is regulated by the Ca^2+^-ATPase, Na^+^/Ca^2+^-exchanger, and Ca^2+^-transporter [23]. Many organelles, such as the autophagosomes, endoplasmic reticulum, endosomes, Golgi apparatus, lysosomes, mitochondria, nucleus, and secretory vesicles, serve as calcium stores involved in calcium signaling/homeostasis by storing or releasing Ca^2+^ [24]. The release and refilling of calcium stores are modulated by many Ca^2+^ channels, pumps, transporters, and Ca^2+^ binding proteins located in or coupled with calcium stores. The IP3R is one of the important receptor channels involved in the regulation of intracellular calcium stores. The IP3R is ubiquitously expressed in most cell types and evokes cellular processes such as embryonic development, gluconeogenesis and neuronal plasticity by controlling Ca^2+^ signals [25]. Expression profiles and pharmacological evidence suggest that IP3R may have important roles in spermatogenesis [7]. However, there is no comprehensive or in-depth literature available on IP3R in male reproduction, especially in the regulation of spermatogenesis and mature sperm function. Therefore, this review will systematically summarize the structural characteristics of the IP3R channel and its expression and function in the male reproductive system, and further discuss future research and strategies for understanding the role of IP3R in the regulation of spermatogenesis and sperm function.

## 2. Structure and Function of the IP3R Channel

**Figure 1:**
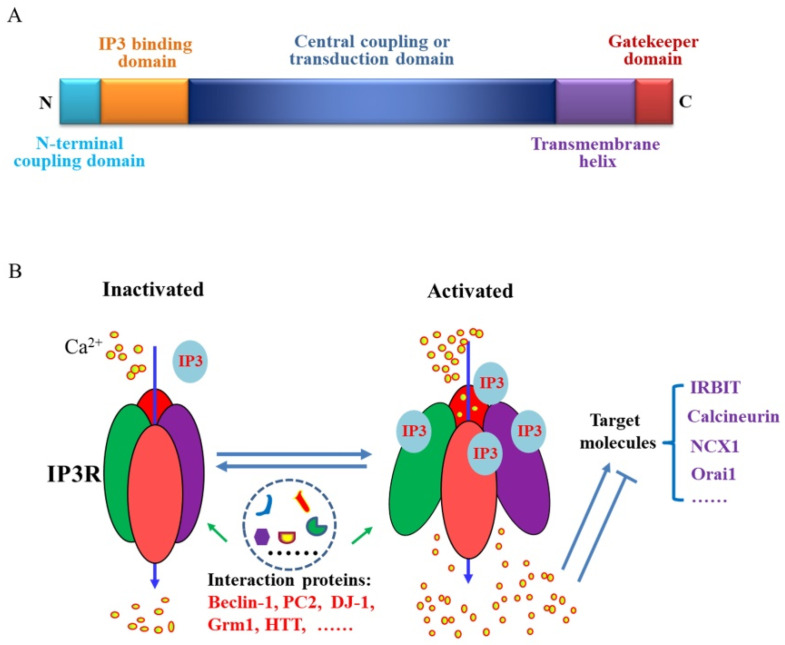
Diagram of the structure and regulation of the inositol 1,4,5-trisphosphate receptor (IP3R) channel. (**A**) Five domains of IP3R. (**B**) Regulation of IP3R by IP3, Ca^2+^ and interaction proteins.

The IP3R is a calcium ion channel located in the endoplasmic reticulum and sarcoplasmic reticulum. It is a tetrameric protein receptor channel comprising four glycoproteins and has a relative molecular weight of about 260 kDa. To date, three types of IP3R channels, IP3R1, IP3R2, and IP3R3, have been identified, and all are encoded by three different genes [26]. A schematic diagram of IP3R proteins is shown in Figure 1A. Five domains found in the three types of IP3R channels include a coupling or inhibiting domain in the N-terminal region, an IP3-binding domain, a central coupling or transduction domain, a six-transmembrane helix, and a carboxy-terminal gatekeeper domain [26]. The binding domains and calcium channel domains of the three types of IP3R channels are highly homologous. The sequence identities between Type I and Type II, and between Type III and Type I, are 69% and 64%, respectively [27]. Because of this high homology, functional heterotetramer channels can be formed between all the different subtypes [28]. Thus, diverse IP3R channels can take part in the regulation of wide ranging physiological activities [29]. At present, the structures of IP3R1 and IP3R3 have been established by cryo-electron microscopy. Resting IP3R1 and IP3R3 have similar structures but different mechanisms of activation and regulation [30,31]. The structure of activated IP3R1, after combining with inositol 1,4,5-trisphosphate (IP3), remains unclear. The IP3R subtypes have diverse mechanisms for sensing stimuli from different signals and for regulating different interacting molecules. Although the affinities of the IP3-binding core of three IP3R isoforms for IP3 are similar, there are some differences in sensitivity towards IP3 (IP3R2 > IP3R1 > IP3R3) and towards the various regulatory factors and proteins [29,32]. The sensitivity to cytosolic Ca^2+^ also varies between the IP3R isoforms. IP3R3 is the most sensitive to modulation by Ca^2+^, followed by IP3R2, then IP3R1 [33]. ATP enhances Ca^2+^ release from IP3R channels. However, the three IP3R isoforms are reported to respond to ATP with differing sensitivities. Knockout (KO) studies in cells showed that Ca^2+^ release through IP3R1 was positively regulated at lower ATP concentrations than IP3R3, and IP3R2 was the isoform most sensitive to stimulatory ATP concentrations [34]. In addition, the sulfhydryl-reagent thimerosal increased the sensitivity of IP3-evoked Ca^2+^ release via IP3R1 and IP3R2 but inhibited IP3R3 activity [35].

IP3R channel activity is mainly regulated by IP3, Ca^2+^ and interaction proteins (Figure 1B). The classical IP3R-mediated calcium signaling pathway is as follows: phospholipase C (PLC) catalyzes the hydrolysis of phosphatidylinositol 4,5-bisphosphate to form IP3 and diacylglycerol. IP3 diffuses into the cytoplasm and binds to the IP3R receptor on the sarcoplasmic or endoplasmic reticulum to regulate the opening of the channel [36]. A low concentration of Ca^2+^ stimulates the opening of the IP3R channel, but a high Ca^2+^ concentration causes closure of the channel, which is known as Ca^2+^-induced Ca^2+^ release. In this process, calcium ions released from the endoplasmic reticulum are further amplified by other Ca^2+^ receptor channels, such as ryanodine receptors. To restore the level of Ca^2+^ in the endoplasmic reticulum or sarcoplasmic reticulum, Ca^2+^ ATPases (SERCA, Sarco- or Endoplasmic Reticulum Ca^2+^ ATPases) pump calcium ions in the cytoplasm back to the endoplasmic reticulum, and thus periodically regulate the cellular transmission of Ca^2+^ to participate in the regulation of various physiological activities [26]. In addition, many interaction proteins and other factors also modulate IP3R’s activity to cooperate with calcium signaling in different ways. This will be discussed in Section 4.

## 3. Expression of IP3Rs in Male Reproduction

**Figure 2:**
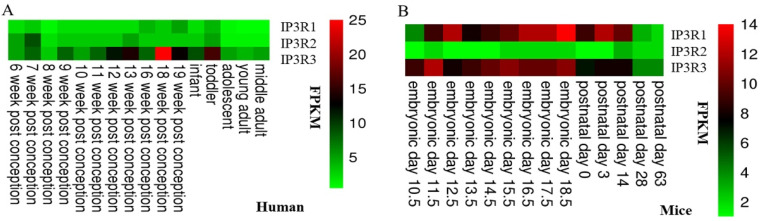
mRNA expression levels of inositol 1,4,5-trisphosphate receptors (IP3Rs) in human (**A**) and mouse (**B**) testes at different stages of development. The data were obtained from the ArrayExpress database (E-MTAB-6814 and E-MTAB-6798). Fragments Per Kilobase Million (FPKM) values from RNA-seq experiments were used to indicate the mRNA expression levels of IP3Rs.

**Figure 3:**
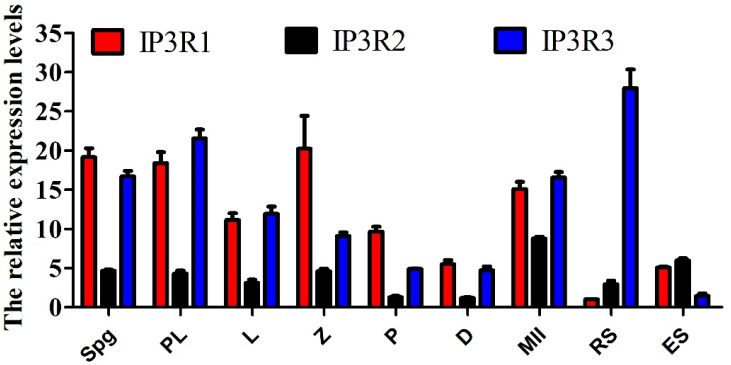
mRNA expression levels of inositol 1,4,5-trisphosphate receptors (IP3Rs) in mouse spermatogenic cells. Spermatogonia (Spg), preleptotene (PL), leptotene (L), zygotene (Z), pachytene (P), diplotene (D), secondary spermatocytes (MII), round spermatids (RSs), and elongated spermatids (ES) were sorted by flow cytometry with Hochest33342/propidium staining. Total RNA was used to analyze mRNA expression levels of IP3R1, IP3R2 and IP3R3 by qPCR. β-actin served as an internal control to normalize the gene expression levels.

Three types of IP3R channel proteins are widely and differently expressed in various tissues, possibly because distinct tissues need different types of functional IP3R receptors. All three types of IP3R are expressed in the testis, spermatogenic cells, and mature sperm [37,38]. The expression of IP3R mRNA in human and mouse testes at different stages of development are depicted in Figure 2. A study in CD1 mice found that the three IP3R mRNAs were expressed in all stages of spermatocyte development [7]. IP3R protein expression exhibited a specific temporal distribution pattern, in spermatogenic cells, with expression dispersed in the cytoplasm in the early stage; then, during sperm differentiation, IP3R protein is gradually expressed on the Golgi complex [7]. Our data also displayed that IP3R1, IP3R2, and IP3R3 mRNAs were expressed in spermatogenic cells of C57/BL6 mice. In early spermatogenic cells, IP3R1 and IP3R3 had higher expression levels than IP3R2 (Figure 3). The expression of IP3R was also detected in the mature sperm of boars and humans [39], and cattle [40]. IP3R was reported to be localized in the acrosome and the principal piece of the flagellum in mature sperm of mice, but the precise location of IP3Rs in sperm remains controversial because of inconsistent results in different studies [7], and the precise subcellular location of IP3R in sperm remains to be determined. The expression of IP3R in human sperm varies slightly in different studies, possibly because of differences in the detecting antibodies. Human sperm may only contain IP3R1 and IP3R3. In sea urchin sperm, IP3R1 protein was also detected in the plasma membrane [41]. In general, most studies suggest that IP3R exists in the acrosome, principal piece of the flagellum, and cytoplasmic droplets in sperm [42,43]. Other research confirmed the presence of IP3R in the acrosome of human sperm [44]. Many organelles, such as the endoplasmic reticulum, Golgi apparatus, and lysosomes, provide the main Ca^2+^ stores in somatic cells. However, mature sperm lack these organelles and the mitochondria and some of the membranous structures of sperm contain many calcium regulatory factors (IP3R and SERCA), which may act to regulate Ca^2+^ stores. It is well known that the acrosome and mitochondria provide the prominent intracellular Ca^2+^ stores in mature sperm [1]. The expression level of IP3R1 decreases after the acrosome reaction, while there is no significant change in the level of IP3R3 [45]. The residual IP3R3 in the acrosome suggests that it may also play an essential role in fertilization and early embryonic development. In addition, an [^3^H] isotope labeling experiment showed that there was a stoichiometric interaction between IP3 and IP3R in sperm before and after the acrosome reaction [45]. IP3R acts as a “bridge” contributing to endoplasmic reticulum–mitochondrial contacts and Ca^2+^ transfer from the endoplasmic reticulum to mitochondria [46]. However, the expression and roles of IP3R in sperm mitochondria are still unknown. The redundant nuclear envelope (RNE), surrounding the axoneme at the base of the flagellum, provides the third Ca^2+^ store in sperm involved in Ca^2+^ mobilization [47]. Preliminary experimental evidence suggests that the RNE contains IP3R, which may regulate sperm hyperactivated motility [42].

## 4. Roles of IP3R in Male Reproduction

There has been limited research on the roles of IP3R in male reproduction; however, previous studies indicated that IP3R may play a key role in spermatogenesis. Some studies found that the inhibition of IP3R1 expression using specific oligonucleotides completely blocked spermatocyte proliferation in mice [36]. In *Caenorhabditis elegans*, IP3R mutant males produced normal sperm but were defective in sperm transfer and spicule insertion, which resulted in infertility [48]. Research in drosophila found that IP3R was required for spermatocyte division, because the IP3R antagonist 2-APB (2-aminoethoxydiphenyl borate) significantly impaired cleavage furrow stability in cytokinesis [49]. Interestingly, 2-APB also increased CatSper-mediated calcium signaling in human sperm [50]. Thapsigargin is a selective inhibitor of Ca^2+^ ATPase and induces the release of calcium ions from IP3-sensitive calcium stores. Thapsigargin significantly induced the acrosome reaction in sperm from hamsters, humans, and mice [51,52,53]. IP3R is thought to be involved in sperm hyperactivation [42,54] and the regulation of thermotaxis [55]. A recent study using a novel permeabilization tool, BioPORTER, to deliver IP3R antibody or IP3 to block or activate IP3R activity, respectively, showed that the IP3R1 channel was involved in the zona pellucida- and progesterone-induced acrosome reaction and calcium influx [56]. These pharmacological or in vitro findings indicated that the IP3R channel is vital for the regulation of spermatogenesis and mature sperm function. In addition, some factors of the IP3R signaling pathway combined with PLCζ may induce egg activation [57].

Research has shown that most IP3R1-deficient mice die in utero at an early stage of embryonic development. The IP3R1-deficient animals surviving to birth suffer from severe ataxia and seizures, and do not survive after the weaning period [58,59]. Phenotypic analysis of the reproductive system showed that epididymal development was significantly impaired (decreased weight) (International Mouse Phenotyping Consortium). Conversely, IP3R2 and IP3R3 knockout mice usually survive, and the fertility of male mice was not remarkably affected [60]. However, it is unclear whether calcium signaling was abnormal in spermatogenetic cells and mature sperm in these KO mice. The knockout of IP3R2 or IP3R3 alone does not affect spermatogenesis or sperm function. Because of the importance of the IP3R channel in various biological processes, it is possible that a specific mechanism of rescuing or compensation was established from other calcium regulation channels. In addition, functional heterotetrameric channels can be formed via the high homology among the three subtypes, which leads to possible compensation from other types of IP3Rs. These possibilities should be verified by models of conditional double or triple KOs of IP3Rs. A recent study demonstrated that IP3R1 located on tubulobulbar complexes, at the junction of the endoplasmic reticulum and plasma membrane, plays an important role in spermatogenesis by regulating Ca^2+^ signals [37,61,62]. Furthermore, the knockdown of IP3R1 in Sertoli cells gave rise to the abnormal morphology of actin protein at the junction of the tube-filled complex membrane and the endoplasmic reticulum and later stage sperm [63,64], indicating that IP3R in Sertoli cells participates in the development of tubulobulbar complexes via regulation of actin dynamics, and also indirectly regulates spermatogenesis.

As the hub of calcium signal regulation, IP3R is regulated by upstream stimulating factors or interacting molecules [65], and it also transmits signals to regulate various downstream biological processes. IP3 and many IP3 analogs can activate the IP3R channel. For instance, adenophostins have a stronger affinity for the IP3R channel receptors than IP3 itself [66]. However, 2-O-IP3, another synthetic analog of IP3, showed a weaker effect on IP3R activation than IP3 [67]. As mentioned above, 2-APB is an inhibitor of IP3R channels [68]. Xestospongin C is a reversible and nonspecific IP3R antagonist inhibiting the IP3-induced Ca^2+^ increase [39]. These inhibitors or agonists can be applied as a tool to determine the roles of the IP3R channel in calcium signaling. Many intracellular molecules, including cAMP [69], NADH [70], and reactive oxygen species (ROS) [71], also modulate IR3R channel activity. In addition, IP3Rs can be regulated by post-translational modification, such as phosphorylation, ubiquitination [72], and transglutaminase-mediated subunit configurations [73]. Nevertheless, how these molecules and post-translational modifications regulate IP3Rs actions in spermatogenesis remains unknown. Furthermore, as mature sperm lack most active transcriptional and translational machinery, the gene expression that normally mediates in vivo functional modulation no longer represents a key factor. Ca^2+^, cAMP, ROS and post-translational modification of proteins play essential roles in the modulation of a range of physiological activities that are critical for sperm function. Hence, mature sperm provide a good model to investigate the regulatory mechanisms of IP3Rs independent of transcription and translation pathways. Recent studies show the involvement of noncoding RNAs in the IP3R regulatory network and possibly Ca^2+^ signaling. miR-240 negatively regulates IP3R1 expression [74]. The inhibition or KO of miR-204 increased IP3R1 levels. The lncRNA eosinophil granule ontogeny transcript (EGOT) upregulated IP3R1 levels via formation of a pre-ITPR1/EGOT dsRNA that induced accumulation and enhanced alternative splicing of IP3R1 primary RNA [75]. Furthermore, a large number of miRNAs and lncRNAs are exclusively or highly expressed in the testes and related to spermatogenesis and even male infertility [76,77,78,79,80]. However, to the best of our knowledge, whether these lncRNAs take part in IP3Rs-mediated Ca^2+^ signal transduction in male reproduction remains undetermined.

To date, more than 100 proteins that interact with IP3R have been found [26]. This diverse set of proteins allows the production of unique spatiotemporal patterns in Ca^2+^ intracellular signaling to respond to various upstream stimulation factors or different downstream target molecules or events. Such proteins can manage intracellular calcium signals through inhibiting or activating the function of the IP3R channel, transmitting downstream signals to regulate gene expression via interaction proteins, or affecting the distribution of IP3R protein for a cascade amplification effect for weakly stimulated calcium signals [25]. Interaction molecules and upstream/downstream regulatory signals should be further studied to clarify the function of the IP3R channel and associated mechanism of regulation. Some reports showed that IP3R channel interaction proteins are essential in spermatogenesis and the regulation of sperm function, which provides a potential alternative process for IP3R to indirectly affect male reproduction. For example, the loss of IP3R interaction protein TMEM203 disrupted intracellular calcium signals and severely affected spermatogenesis, which resulted in the absence of mature sperm production and sterility [81]. Fyn tyrosine kinase can bind to IP3R and cause phosphorylation at tyrosine 353, which in turn leads to increased sensitivity of the IP3R channel and promotes a long-term increase in calcium signaling [82]. Male mice remained fertile after the loss of Fyn; however, testicular development was abnormal and the quantity of sperm was markedly reduced [83]. The IP3R interaction proteins known to affect male reproduction are listed in Table 1. However, further studies are needed to investigate the interplay between IP3Rs and these proteins in reproductive cells that cause impaired reproduction. Additionally, the functions of other IP3R interaction proteins in spermatogenesis and mature sperm should be examined to fully understand the role of the IP3R channel in male reproduction.

## 5. Final Remarks

Well-delineated research and substantial evidence, especially in mammals, are still required to fully understand the significant roles that IP3Rs play in spermatogenesis and the regulation of sperm function. The IP3R channel is diverse because of the heterologous formation of functional channels between various subtypes, and distinct subtypes may compensate for the loss of one type of IP3R. Therefore, there may be no apparent reproductive phenotype in gene KO mice of a single type of IP3R. Considering that mice cannot survive after systemic IP3R1 KO, further studies are needed to elucidate the function and mechanism of the IP3R channel in the reproductive system through tissue-specific KO animal models of IP3R1, and models with specific double or triple KOs of IP3Rs. In the clinical research setting, we should also focus on screening and studying sperm samples with defects in multiple IP3R subtypes. Further investigation into the roles of calcium signaling mediated by this channel in the regulation of mature sperm function will provide insight into the diagnosis and treatment of male infertility. In addition, the crosstalk between IP3R and other Ca^2+^ stores, pumps or interaction factors, should also be investigated to fully uncover the roles and mechanisms of IP3R in male reproduction. 

## Figures and Tables

**Table 1:** Effect of inositol 1,4,5-trisphosphate receptor (IP3R) interaction proteins on male fertility. AZS, asthenozoospermia.

Protein	Relationship with IP3	Functions in Male Reproduction
CDK1	Phosphorylates IP3R1 and increases IP3 binding [25]	Small testes, azoospermia, arrest of male meiosis, and infertility [84]
Beclin-1	Forms interaction complex [85]	Overexpression attenuated the impairment of spermatogenesis [86]
CIB1	Activating and inhibitory protein ligand of the IP3R [87,88]	Azoospermia, abnormal spermiogenesis, and infertility [89]
PKA	Phosphorylates IP3R [69]	AZS and infertility [90]
PKG	Phosphorylates IP3R [69]	Abnormal penile erection and reduced male fertility [91]
Akt1	Phosphorylates IP3R [92]	Abnormal spermatogenesis [93]
HTT	Forms an interaction complex and enhance IP3R activity [94]	Oligozoospermia, small testes, and subfertility [95]
BRCA1	Sensitizes the IP3R to its ligand [96]	Spermatogenesis arrest and infertility [97]
Grm1	Forms a physical tether linking [25]	Infertility [98]
PLC-β	Forms a interaction complex, enhances IP3R activity and delivers IP [99]	Subfertility [100]
Orai1	Acts as downstream effectors [101]	Deficiencies in elongating spermatids and fertility [102]
PC2	Forms complexes with IP3Rs [103]	Infertility [104]
DJ-1	Interacts with the IP3R [105]	Related with AS [106] and polymorphisms associated with infertility [107]

## Abbreviations

IP3R	Inositol 1,4,5-trisphosphate receptor
PIP2	Phosphatidylinositol 4,5-bisphosphate
PLC	Phospholipase C
2-APB	2-aminoethoxydiphenyl borate
CDK1	Cyclin-dependent kinase 1
PKA	Protein kinase A
PKG	Protein kinase G
CIB1	Calcium and integrin binding 1
HTT	Huntingtin
BRCA1	Breast cancer 1, early onset
cAMP	Cyclic adenosine monophosphate
ATP	Adenosine triphosphate
TBCs	Tubulobulbar complexes
PC2	Polycystin 2
AZS	Asthenozoospermia
